# Heteroplasmy concordance between mitochondrial DNA and RNA

**DOI:** 10.1038/s41598-019-49279-7

**Published:** 2019-09-10

**Authors:** Ruoyu Zhang, Kiichi Nakahira, Augustine M. K. Choi, Zhenglong Gu

**Affiliations:** 1 Division of Nutritional Sciences, Cornell University, Ithaca, New York 14853 USA; 2 Division of Pulmonary and Critical Care Medicine, Joan and Sanford I. Weill Department of Medicine, Weill Cornell Medicine, New York, NY 10065 USA; 3 Department of Pharmacology, Nara Medical University, Kashihara-shi, Nara Japan; 4 Present Address: Regeneron Pharmaceuticals, Inc, Tarrytown, NY 10591 USA

**Keywords:** RNA sequencing, Next-generation sequencing, DNA sequencing

## Abstract

Mitochondrial DNA (mtDNA) heteroplasmies are associated with various diseases but the transmission of heteroplasmy from mtDNA to mitochondrial RNA (mtRNA) remains unclear. We compared heteroplasmies in mtRNA from 446 human B-lymphoblastoid cell lines to their corresponding mtDNA using deep sequencing data from two independent studies. We observed 2786 heteroplasmies presenting in both DNA and RNA at 1% frequency cutoff. Among them, the frequencies of 2427 (87.1%) heteroplasmies were highly consistent (less than 5% frequency difference) between DNA and RNA. To validate these frequency consistencies, we isolated DNA and RNA simultaneously from GM12282 cell line used in those two sequencing studies, and resequenced its heteroplasmy sites. Interestingly, we also observed the rapid changes of heteroplasmy frequencies during 4 weeks of the cell culture: the frequencies at Day 14 increased by >25% than those at Day 0. However, the heteroplasmy frequencies from the same time point were highly consistent. In summary, our analysis on public data together with *in vitro* study indicates that the heteroplasmies in DNA can be transcribed into RNA with high fidelity. Meanwhile, the observed rapid-changing heteroplasmy frequency can potentially disturb cell functions, which could be an overlooked confounding factor in cell line related studies.

## Introduction

The genetic information from DNA needs to be transcribed to RNA for protein synthesis to achieve biological functions. The fidelity of the sequences information from DNA to RNA is critical since the inconsistency introduced during the transcription process may contribute to genetic variations, and further affect protein synthesis and/or the gene expression level^1^. Studies has shown that there are widespread RNA and DNA differences (RDD) in human nuclear genome^2,3^, however, the knowledge of mitochondrial RDD is still limited.

In contrast to only two copies of nuclear DNA (nDNA), there can be hundreds to thousands copies of mitochondrial DNA (mtDNA) existing within a single eukaryotic cell. The nature of multiple copies of mtDNA and their highly variable sequences make it possible that mutated mtDNA can co-exist with wild type mtDNA at any ratio, which is termed as heteroplasmy^4^. Recently, there are many studies indicating that mitochondrial heteroplasmic mutations are associated with broad spectrum of human diseases such as muscular dystrophy, diabetes, Alzheimer’s diseases and cancer^4–12^. In addition, the allele frequency of a deleterious mutation would be critical for its pathogenicity^13^. Nevertheless, there are few studies to investigate the transmission of mtDNA heteroplasmies to mitochondrial RNA (mtRNA). It remains unclear whether mutated and wild type mtDNA are transcribed proportionally.

Advances in sequencing technologies (i.e., Next Generation Sequence) enable us to study heteroplasmy at single nucleotide resolution. By comparing the sequence of mitochondrial RNA and DNA from the same individual, we are able assess the RDDs in mitochondrial genome. In this study, we comprehensively analyzed 446 pairs of RNA and DNA sequences from human lymphoblastoid cell lines, which were a subset of individuals from 1000 genome project^14,15^. We investigated whether the extensively existing heteroplasmic variations in mtDNA were also observable in mtRNA, moreover, whether the heteroplasmy frequencies presented in RNA would keep consistent with that in DNA. Our results demonstrated that the majority of heteroplasmies in mitochondrial DNA (mtDNA) could be transcribed proportionally to RNA regardless of their pathogenic degrees. We further experimentally validate this observation by sequencing DNA and RNA extracted simultaneously from the same cells and confirmed that there was minimal difference in heteroplasmies between DNA and RNA. Interestingly, during the cell culture process, we observed that the heteroplasmies frequencies were changing dramatically with time which may disturb the cellular functions according to the mtDNA heteroplasmy threshold effects.

## Results

### Identification of heteroplasmies using DNA and RNA sequencing data

To assess the RNA-DNA difference in mitochondrial genome, we first comprehensively analyzed 446 pairs of RNA and DNA sequences from immortalized human lymphoblastoid cell lines derived from individuals from a variety of human populations. RNA-seq data of these were retrieved from Geuvadis RNA-seq Project^15^. These individuals were a subset of 1000 Genome Project, thus the DNA-seq data from the same individual were available from 1000 Genome Project^14^. To investigate mitochondrial heteroplasmy, the sequencing reads were first aligned to human reference genome hg19, and after a series of quality control steps, we retained high quality mtDNA sequencing reads for subsequent mitochondrial heteroplasmy analysis (Methods). We examined two statistics of the mitochondrial genome alignment results: median sequencing coverage and average cover rate of the mitochondrial genome (Fig. S1A,B). The median sequencing coverage of mtDNA and mtRNA were 2077 and 4557 for these samples, respectively. Although the overall sequencing coverage of mtDNA was lower than that of mtRNA, the distribution was more uniform across the entire mitochondrial genome in DNA (Fig. S1C). It’s not surprising that mtRNA had fluctuating coverage distribution as the coverage was highly affected by the gene expression level of each mitochondrial gene. The mitochondrial genome cover rate was calculated as the percentage of mitochondrial genome positions with sequencing coverage >200. The average cover rates in DNA and RNA-seq data were 99.76% and 97.09%, respectively. These sequencing coverage and mitochondrial genome cover rate were sufficient to systemically investigate heteroplasmy at 1% minor allele frequency (MAF) cutoff.

The details of heteroplasmy identification procedure can be found in Methods part. Briefly, we first applied relative conservative criteria to identify heteroplasmy in DNA and RNA sequencing data, separately, at 1% MAF cutoff. We then integrated the results from both DNA and RNA sides: once we identified a specific site as a heteroplasmy site in either DNA or RNA data, the criteria to screen heteroplasmy signal from the other data source was lowered to rescue the sites with lower coverage (Methods). All identified heteroplasmies were categorized into “Observed in both DNA and RNA” (BDR), ‘Observed Only in DNA” (OD) and “Observed Only in RNA” (OR) groups. We identified 2786 heteroplasmies presenting in both DNA and RNA in total, 219 only found in DNA and 682 only in RNA, respectively. Consistent with previous studies^16^, majority of the heteroplasmies have low frequencies. In DNA-seq data, 77.5% heteroplasmy has frequency <5%, and 86.8% has frequency <10%.

### Compare heteroplasmy frequencies between DNA and RNA

**Figure 1:**
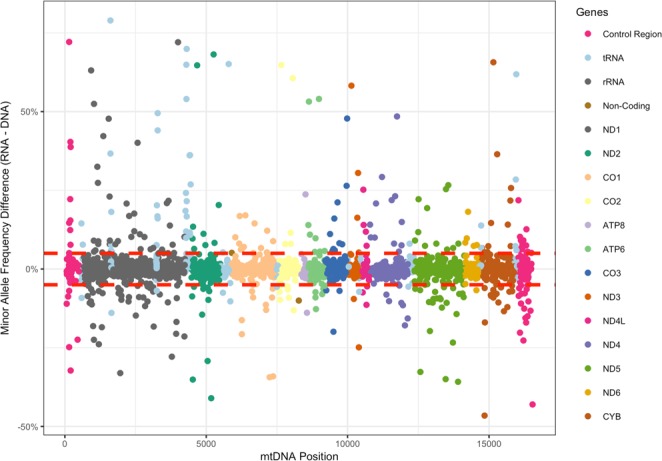
Heteroplasmy frequency difference between DNA and RNA. Each point is a heteroplasmy sites. X-axis is the mtDNA coordinates and Y-axis is the frequency difference calculated as (RNA-DNA), thus a positive number indicates that frequency in RNA is higher than that in DNA, and negative number indicates that frequency in RNA is lower than that in DNA. The region between the two red dashed lines indicates frequency difference <5%, 87.1% points fall into this region.

According to the heteroplasmy threshold effects theory^12^, heteroplasmy frequencies could be a determinant of the deleterious mutations. We first investigated whether the heteroplasmic mutations in DNA can be transcribed into RNA proportionally. Using these datasets, we compared the heteroplasmy frequency differences between DNA and RNA. For each heteroplasmy in BDR group, we computed its MAF in DNA, then computed the frequency of the same allele in RNA. The frequency differences were calculated as RNA-frequency minus DNA-frequency (RNA-DNA). The heteroplasmy frequencies were highly consistent between DNA and RNA (87.1% of the heteroplasmies had frequency difference <5%, and 93.6% had frequency difference <10%) (Fig. 1), indicating that most of the heteroplasmy information can be transmitted faithfully from DNA to RNA. We also observed that some heteroplasmies had noticeable frequency difference from DNA to RNA (3.0% of the heteroplasmies had frequency difference >20% and 1.6% had frequency difference >30%). Among these heteroplasmies, there were heteroplasmies having higher frequencies in DNA than RNA. One possible explanation for the heteroplasmies with high frequencies in DNA but low frequencies in RNA (HDLR heteroplasmy) is that the mutation transmission from DNA to RNA are repressed due to the deleteriousness of these mutation. To test this hypothesis, we annotated the heteroplasmies with Combined Annotation Dependent Depletion (CADD) scores^17^, as a measurement of their pathogenic potentials. We compared the CADD scores of HDLR heteroplasmies (DNA frequency at least 5% greater than RNA frequency) to the rest of heteroplasmies. However, the CADD scores of the HDLR heteroplasmies were not significantly higher than those of non-HDLR heteroplasmies (one-side t-test, ***P value*** = 0.2276, Fig. S2). We next tested whether the HDLR heteroplasmies were more like to be diseases-associated by chi-squared test, but the result was also not significant (***P value*** = 0.6877). In addition, we also identified 219 heteroplasmies in OD group. There was no significant difference between the CADD scores of the heteroplasmies observed in OD group and those of BDR group (one-side t-test, ***P value*** = 0.1). The average heteroplasmy frequency of OD group was 2.6%, and more than half of these heteroplasmies had frequency <1.5%, it’s possible that these heteroplasmies were dropouts in the RNA-seq data. Thus, we didn’t find the evidence for negative selections of heteroplasmy transmission from DNA to RNA.

### Rapid heteroplasmy frequency changes during cell culture

**Figure 2:**
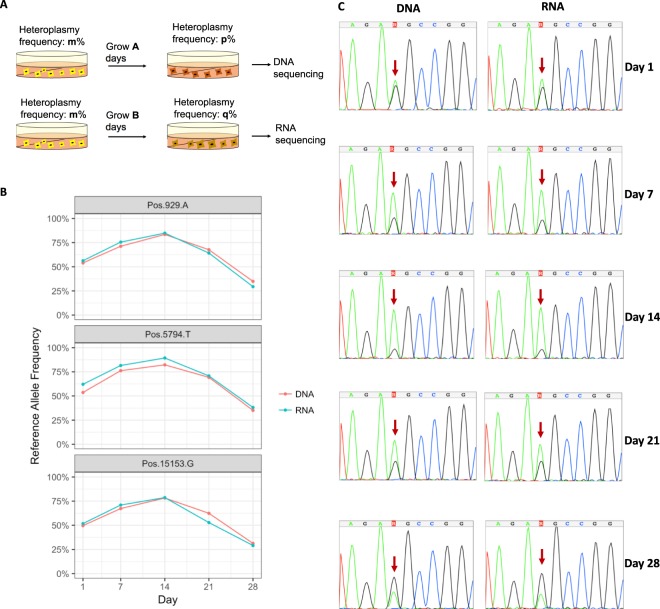
Rapid heteroplasmy frequency changes during cell growing process. (**A**) Illustration of how heteroplasmy dynamics can cause artificial frequency difference between DNA and RNA. If cells used for DNA and RNA sequencing were cultured independently. At day 0, the heteroplasmy frequencies of both cell cultures were m%. Cells for DNA sequencing were grown for A days and the heteroplasmy frequency changed to p% while cells for RNA sequencing were grown for B days and heteroplasmy frequency changed to q%. In the sequencing data, the RNA-DNA heteroplasmy frequency difference would be q%-p%, which cannot reflect the real RNA-DNA frequency consistency. (**B**) Three heteroplasmy frequencies in both DNA and RNA were tracked in 28 days. In all three heteroplasmies, the frequencies increased from Day 1 to Day 14 and decreased afterwards. But at same time point, the frequencies of DNA and RNA were very close. (**C**) Sanger sequencing traces of heteroplasmy site 929 A > G in DNA and RNA at different time point. Red arrow indicated the heteroplasmy site. See also Fig. S3 for the other two heteroplasmy sites.

In the above analysis, DNA and RNA sequencing data were retrieved from two independent large-scale sequencing projects using the same cell lines (1000 Genome Project and Geuvadis RNA-seq Project), suggesting that the cell cultures for DNA and RNA extraction were performed independently. Assuming that the heteroplasmy frequencies could change during the cell culture period, and that DNA and RNA were extracted at different time points, it is possible that heteroplasmy frequencies between DNA-seq and RNA-seq data are different. To test this hypothesis, we sought to analyze three heteroplasmy sites with |DNA-RNA| frequency differences >45% from the above analysis (Table 1) in the cell line GM12282, one of cell lines that were used in the two sequencing projects. We grew this cell line for 28 consecutive days and extracted DNA and RNA simultaneously from the same cells every 7 days to compare DNA and RNA heteroplasmy frequency (Fig. 2A and Methods). We also tracked the heteroplasmy frequency dynamics of these three heteroplasmy sites over the period of cell culture (28 days). Our data showed that heteroplasmy frequency kept high consistency between DNA and RNA at each time point (Fig. 2B,C**)**. For example, the A allele frequency difference of site 929 was 63.1% (DNA 20.2%, RNA 83.3%) in the public dataset. In contrast, from our experiment data, the difference was only 2.2% (DNA 54.0%, RNA 56.2%) at day 0 and 1.4% (DNA 83.4%, RNA 84.8%) at Day 14. Meanwhile, we also observed dramatic frequency changes in all the three heteroplasmy sites during the 28 days (Fig. 2B,C). For example, at heteroplasmy site 15153 G > A, the frequencies of G allele were 49.6% (DNA) and 51.7% (RNA) at Day 0. The frequency increased to 78.2% (DNA) and 78.7% at Day 14, and then decreased to 31.2% (DNA) and 29.0% (RNA) at Day 28. We noticed that all of the three heteroplasmies presented the similar patterns of heteroplasmy frequency change during the 28 days, and also that they had quite similar frequencies between DNA and RNA at the same time point, (Fig. 2B,C). This observation suggested that these three mutations could locate at same mtDNA copies and therefore were replicated and transcribed together.

**Table 1:** Heterolasmy sites with noticeable frequency difference between DNA-seq and RNA-seq data in the cell line GM12282.

mtDNA position	Reference allele	Alternative allele	Reference frequency in DNA	Reference frequency in RNA	Frequency difference	Annotation	CADD score
929	A	G	20.2%	83.3%	63.1%	12S RNA	10.54
5794	T	C	21.1%	86.2%	65.1%	tRNA-Cys	10.15
15153	G	A	19.7%	85.4%	65.7%	CYB (NS)	23.5

Finally, we independently repeated 28-days cell culture using the same batch of cell stocks to address the reproducibility of our results. At Day 28, we observed the heteroplasmy frequency changes comparing to Day 0 in all three experiment repeats (Table 2). For instance, A allele frequencies of site 929 at Day 28 were 44.4%, 81.6%, 57.5% in three independent cell culture experiments, respectively. The observation that the same heteroplasmy site can have different frequencies among three independent experiments also supports our results that heteroplasmy frequency can be changed when cells are grown at the different times. Taken these results together, our *in vitro* study suggests that heteroplasmy changes rapidly within 2 weeks of cell culture, which can be transmitted to RNA with high fidelity.

**Table 2:** Reference allele frequencies of the three heteroplasmy sites in cell line GM12282 at day 28 of  in three experiment repeats.

mtDNA postion	Reference allele	Alternative allele	Reference allele frequency at Day 28	
929	A	G	Repeat 1	44.4%
			Repeat 2	81.6%
			Repeat 3	57.5%
5794	T	C	Repeat 1	43.6%
			Repeat 2	80.7%
			Repeat 3	58.1%
15153	G	A	Repeat 1	39.7%
			Repeat 2	77.1%
			Repeat 3	52.9%

## Discussion

Mitochondria are indispensable organelles in eukaryotic cells. Its primary function is converting nutrients and oxygen to ATP by oxidative phosphorylation (OXPHOS) to support various cellular functions. Besides, mitochondria also participate in diverse functions such as calcium signaling, autophagy and apoptosis^18,19^. Unique from other organelles, mitochondria host their own genome: mtDNA, which encodes 13 protein subunits essential for OXPHOS and an RNA machinery (2 rRNAs and 22 tRNAs) for mtDNA translation. mtDNA is highly susceptible to mutations, since its DNA replication and repair mechanism was not as robust as nuclear DNA^20^. Recent studies reported that mtDNA heteroplasmic mutations have high prevalence even in general healthy population, ∼90% of the individuals carry at least one heteroplasmy^16,21^. The deleterious heteroplasmies among them, once reached the threshold frequency, could be a potential source of mitochondrial dysfunction^4,5,22^ if mutations from mtDNA are transcribed into mtRNA for the further protein synthesis. However, the knowledge of how faithfully the mtDNA heteroplasmic mutations could be transcribed into RNA remains still limited and unclear.

In this study, by comparing the paired genomic and transcriptomic data from 446 human lymphoblastoid cell lines, we reported that most heteroplasmies identified at DNA level can be transcribed into RNA. Moreover, the frequency of heteroplasmies were highly consistent between DNA and RNA. For the sites with noticeable frequency differences, we experimentally verified that the differences could be artifacts caused by heteroplasmy dynamics. Our results suggested that there might be minimal negative selection in the mitochondrial transcription step. Therefore, the heteroplasmies, including the pathogenic ones, were able to be passed to RNA. In addition, because of the high similarity between DNA and RNA, mitochondrial RNA sequencing data could be an alternative source to access mitochondrial genome mutation spectrums. However, a limitation of using RNA-seq for mutation identification is that, the sequencing coverages would be highly fluctuated by gene expression levels. In this study, we observed that the thirteen protein coding regions consistently had high coverages, while the mtDNA control regions and tRNA regions had relative low coverages (1000–2000X). Low frequency heteroplasmies within those regions might be missed due to the fluctuated coverage. In the future studies, RNA heteroplasmy studies could emphasize on protein coding regions. We also investigated RNA editing/modification events in mitochondrial genome by inspecting the heteroplasmies only observed in RNA. There are three well-characterized mtRNA editing/modification sites (295, 2617 and 13710) from previous studies^23,24^. In this project we confirmed that most of the individuals has modification/editing at these three sites (Table S1), and the edited allele frequency were varied from individual to individual.

Another important insight of this study is to provide the direct evidence of rapid heteroplasmy frequency changes during the cell cultures. The heteroplasmy threshold effect is for mitochondrial pathology: the same mitochondrial genetic defects can lead to dramatic variability in the disease outcomes, since the phenotypic manifestations will not be triggered until a certain mtDNA heteroplasmy frequency is exceeded the threshold^12^. Therefore, the frequency of a heteroplasmy is a critical factor for its functional impacts. The dynamics of heteroplasmy frequencies were studied in different scenarios in previous studies^25–28^, Lehtinen *et al*. showed that pathogenic heteroplasmy 3243 A > G can change during 4 months if growing in selective medium^28^, and Stewart *et al*. reviewed the possible mechanisms of how a single mtDNA mutation can eventually reach high frequency after multiple rounds of cell divisions^4^. Furthermore, Rahman *et al*. compared the heteroplasmy 3243 A > G frequency in blood samples separated by 9–19 years apart of six Mitochondrial﻿ encephalopathy, lactic acidosis, and stroke-like episodes (MELAS) syndrome patients, and found a decline of the frequency in all the patients^25^. In a recent study, Burgstaller *et al*. compared oocytes as well as somatic heteroplasmy changes in a mouse model, and reported difference in mean heteroplasmy frequencies between mice generations^27^. These studies all suggested that heteroplasmy frequency is not a constant value over time. In a long-term context, the heteroplasmy frequency gradually changes. In this study, we tracked heteroplasmy frequencies in cell line cultures in 28 days. Remarkably, we found that the heteroplasmy frequency had dramatic changes in a short time frame: as much as ~30% within seven days. We also noticed that the frequency changes are not monotonous, for a specific allele, the frequency can move both up and down (Fig. 2B). The three heteroplasmies we tracked here was not reported to be associated with disease before, and the CADD scores of them are 10.54 (929 A > G), 10.15 (5794 T > C) and 23.5 (15153 G > A), respectively. Generally, mutation with CADD score >15 will be considered as high pathogenic potential^17^. In our measurement, high pathogenic potential heteroplasmy 15153 G > A also presented fluctuated frequency during 28 days, and this fluctuation potentially results in different cellular manifestations.

Although *in vitro* study using cell lines is one of the most common experimental methods in a wide range of biological research, there are a number of cases that original data from *in vitro* studies were not able to be reproduced^29^. A recent study suggested genetic and transcriptional evolution in cancer cell lines is one explanation of the variations^29^. The heteroplasmy dynamics we observed in this study could also contribute to the genetic instability of cell lines, which may be a vital but overlooked confounding factor of the studies involving cell lines. Meanwhile, most of the heteroplasmies in this study showed consistent frequencies between DNA and RNA, suggesting that heteroplasmy could also be stable during time. Further studies are need to elucidate the heteroplasmy dynamic/stability. When cell line-based studies are conducted, it might be beneficial to take the heteroplasmy dynamics into account, which could help improve the reproducibility of the cell-line recruiting studies. Future functional studies are still needed to elucidated how these fast changed heteroplasmies contribute to cell functions.

Intriguingly, in this study, the three heteroplasmies kept similar frequency at all time points, suggesting they could be phased at same mtDNA copies. Therefore, there could be two types of mtDNA copies existing in the cells used in our study: one harbors the three mutated alleles, the other one harbors the wild type allele. The frequency changes denoted that these two distinct mtDNA were competing for the majority each other during the cell growing process, and they may have different fitness under different conditions. It’s possible that these two types of mtDNA are host by different cells, therefore forming two distinct cell populations. Different fitness of the mtDNA may contribute to the cell population’s fate. On the other hand, both mtDNA types can also be host by a single cell. In this situation, the competing of these two mtDNA may define the polarization of the cell. Single cell mtDNA sequencing could be performed in the future to investigate this topic.

Our analysis of mitochondrial DNA and RNA sequence data from two independent projects using the same cell line suggests the followings: First, the majority of heteroplasmies in DNA were transcribed proportionally to RNA regardless of their pathogenic degrees. Second, there were also discrepancies of heteroplasmy patterns between DNA and RNA. However, our *in vitro* study demonstrated that the patterns of heteroplasmies from DNA and RNA isolated from the identical cell samples had minimal difference. Importantly, we also observed that the heteroplasmies were changing with time rapidly and randomly during the 4 weeks, which may explain the inconsistency of heteroplasmy patterns from two independent human genome sequencing studies. In conclusion, we showed that heteroplasmy in DNA was passed to mRNA and that heteroplasmy changed rapidly in human cell lines. A caution may need to be taken for studies recruiting cell lines. Further studies are necessary to assess whether or not the cellular heteroplasmy can also change rapidly and randomly in primary cells and *in vivo*, and whether they will further disturb cellular functions.

## Methods

### Data retrieval and pre-processing

DNA and RNA raw sequencing data was retrieved from 1000 Genome Project (http://www.internationalgenome.org/data) and Geuvadis RNA Sequencing Project (http://www.geuvadis.org/web/geuvadis/rnaseq-project), respectively. Totally, there were 446 pairs of DNA and RNA sequences. To retrieve mtDNA candidates reads, raw sequencing was first mapped to the mitochondrial genome. The mapped reads were then re-mapped to the combined human genome, hg19 for the nuclear genome and the revised Cambridge Reference Sequence (rCRS) for the mitochondrial genome with bowtie2^30^. To remove possible contaminations from nuclear mitochondrial sequence (NUMTs), we only retained reads which could be uniquely mapped to mitochondrial genome. Retained reads were further processed with the GATK best practice workflow, including Mark duplicates, Indel realignment, and Base quality score recalibration steps^31^. The sequencing information for each mtDNA sites were compiled with Samtools mpileup function^32^.

### Heteroplasmy identification and annotation

We first applied relative conservative criteria to identify heteroplasmies in DNA and RNA sequencing data separately at 1% minor allele frequency cutoff. The criteria were as followings: 1) Sequencing coverage >200. 2) Minor allele frequency > = 1%. 3) Minor allele must be observed at least twice from each strand. We then integrate heteroplasmy information from both sides to evaluate heteroplasmy concordance between DNA and RNA. To avoid possible artifacts caused sequencing coverage, we only consider sites with depth >200 in both DNA and RNA data. The heteroplasmies were grouped into the following categories: A heteroplasmy was grouped into “Observed in both DNA and RNA” (BDR) if it met any of the followings: 1. Identified as a heteroplasmy at 1% frequency cutoff in both DNA and RNA-seq data of the same individual. 2. If identified as a heteroplasmy only in DNA-seq data, the minor allele of this heteroplasmy needs to have at least 3 sequencing reads to support in the RNA-seq data from the same individual. 3. If identified as a heteroplasmy only in RNA-seq data, the minor allele of this heteroplasmy needs to have at least 3 sequencing reads to support in the DNA-seq data from the same individual. Otherwise, a heteroplasmy would be grouped into “Observed Only in DNA” (OD) and “Observed Only in RNA” (OR) groups if it was identified in either DNA or RNA data.

Heteroplasmies were annotated by customized scripts. Pathogenic potential of variants was predicted using Combined Annotation-Dependent Depletion (CADD) score (version 1.3)^17^. The disease associated mtDNA mutations were obtained from the MITOMAP database^33^.

### Cell culture and point heteroplasmy sequencing

GM12282 B-lymphocyte cell line was purchased from Coriell Institute (https://www.coriell.org/). Cells were grown at 37 °C, 5% CO_2_, in Roswell Park Memorial Institute Medium 1640 with 2 mM L-glutamine and 15% fetal bovine serum with penicillin and streptomycin. Cells used for all experiments in this study were from the same cell aliquots that were prepared in advance and stored in liquid nitrogen. After thawing, cells were grown for 2 days to recover, followed by sub-culture (Day 0). Cell passage was performed every 2–3 days and fresh medium were replaced to maintain cell density with less than 2 × 10^6^ cell/ml. (https://www.atcc.org/~/media/pdfs/culture%20guides/animcellculture_guide.ashx). Cells were then collected and stored at −80C after snap freezing at Day 0, 7, 14, 21 and 28. DNA and RNA were extracted from the collected cells (1 × 10^6^ cells) simultaneously with AllPrep® DNA/RNA Mini Kit (Qiagen, #80204). RNA was reverse transcribed to cDNA with SuperScript™ III First-Strand Synthesis System (Invitrogen, catalog number: 18080051).

### PCR

To amplify mtDNA region including the mutation sites, total DNA was amplified by PCR using Phusion High-Fidelity DNA Polymerase (ThermoFischer Scientific). For assessing mtRNA mutation, extracted total RNA was first treated with ezDNase™ Enzyme (ThermoFisher) to remove DNA contamination. Treated total RNA was converted to single strand cDNA by using High-Capacity cDNA Reverse Transcription Kit (ThermoFischer Scientific) after the incubation with ezDNase, followed by PCR amplification. The following primer sets were used for the amplification of region including mtDNA and mtRNA mutation.

Location 929 forward: GATCTACACTCTTTCCCTACACGACGCTCTTCCGATCTCACGGGAAACAGCAGTGATT, reverse: GTGACTGGAGTTCAGACGTGTGCTCTTCCGATCTATTGGGGAGGGGGTGATCTA;

5794 forward: GATCTACACTCTTTCCCTACACGACGCTCTTCCGATCTTTCAATCTACTTCTCCCGCCG, reverse: GTGACTGGAGTTCAGACGTGTGCTCTTCCGATCTGGGGGTGAGGTAAAATGGCT;

15153 forward: GATCTACACTCTTTCCCTACACGACGCTCTTCCGATCTCAGAAACCTGAAACATCGGCA, reverse: GTGACTGGAGTTCAGACGTGTGCTCTTCCGATCTCCAATGTATGGGATGGCGGA.

### Sanger sequencing

10 ng of PCR products were subject to sequencing using the following primers: Location 929, CACGGGAAACAGCAGTGATT; 5794, TTCAATCTACTTCTCCCGCCG; 15153, CAGAAACCTGAAACATCGGCA. Sanger sequencing data were visualized and allele frequencies were quantified by MutationSurveyor (V5.1.1).

### Ethics statement

The 1000 Genomes Project developed guidelines on ethical considerations for investigators doing sampling. All collections included in the Project followed these ethical guidelines and model informed consent language. The 1000 Genomes Project Steering Committee, with input from the Project’s Samples and ELSI Group, made final decisions about which populations and sample sets to include in the Project.

## Supplementary information

Heteroplasmy concordance between mitochondrial DNA and RNA Supplement

## Data Availability

DNA and RNA raw sequencing data can be retrieved from 1000 Genome Project (http://www.internationalgenome.org/data) and Geuvadis RNA Sequencing Project (http://www.geuvadis.org/web/geuvadis/rnaseq-project), respectively.
