# Supplementary material for: Heteroplasmy concordance between mitochondrial DNA and RNA
Source: Sci Rep. 2019 Sep 10;9:12942. doi: 10.1038/s41598-019-49279-7 (PMC6737107; doi:10.1038/s41598-019-49279-7)
Supplement: Supplementary file 1 — Heteroplasmy concordance between mitochondrial DNA and RNA Supplement [file 41598_2019_49279_MOESM1_ESM.docx]

**Supplement Material**

## **Heteroplasmy concordance between mitochondrial DNA and RNA**

Ruoyu Zhang^1,^^4†^, Kiichi Nakahira^2,3†^, Augustine M.K. Choi^2^, Zhenglong Gu^1^

^1^Division of Nutritional Sciences, Cornell University, Ithaca, New York 14853, USA

^2^Division of Pulmonary and Critical Care Medicine, Joan and Sanford I. Weill Department of Medicine, Weill Cornell Medicine, New York, NY 10065, USA.

*^3^Department of Pharmacology, Nara Medical University, Kashihara-shi, Nara, Japan*

† These authors contributed equally to the work

Address correspondence to:

Zhenglong Gu ([zg27@cornell.edu](mailto:zg27@cornell.edu)), Ruoyu Zhang ([rz253@cornell.deu](mailto:rz253@cornell.deu))

^4^ Present address: Regeneron Pharmaceuticals, Inc, Tarrytown, NY 10591, USA

**Figure S1 mtDNA sequencing statistics in DNA and RNA seq data.** (A) The median sequencing coverage of mtDNA and mtRNA among the 446 samples. (B) The mitochondrial genome cover rate by DNA and RNA seq data. Error bar indicates the standard deviation of across 446 individuals for DNA and RNA, respectively in (A) and (B). (C) The sequencing coverage across the entire mitochondrial genome in DNA and RNA, respectively.


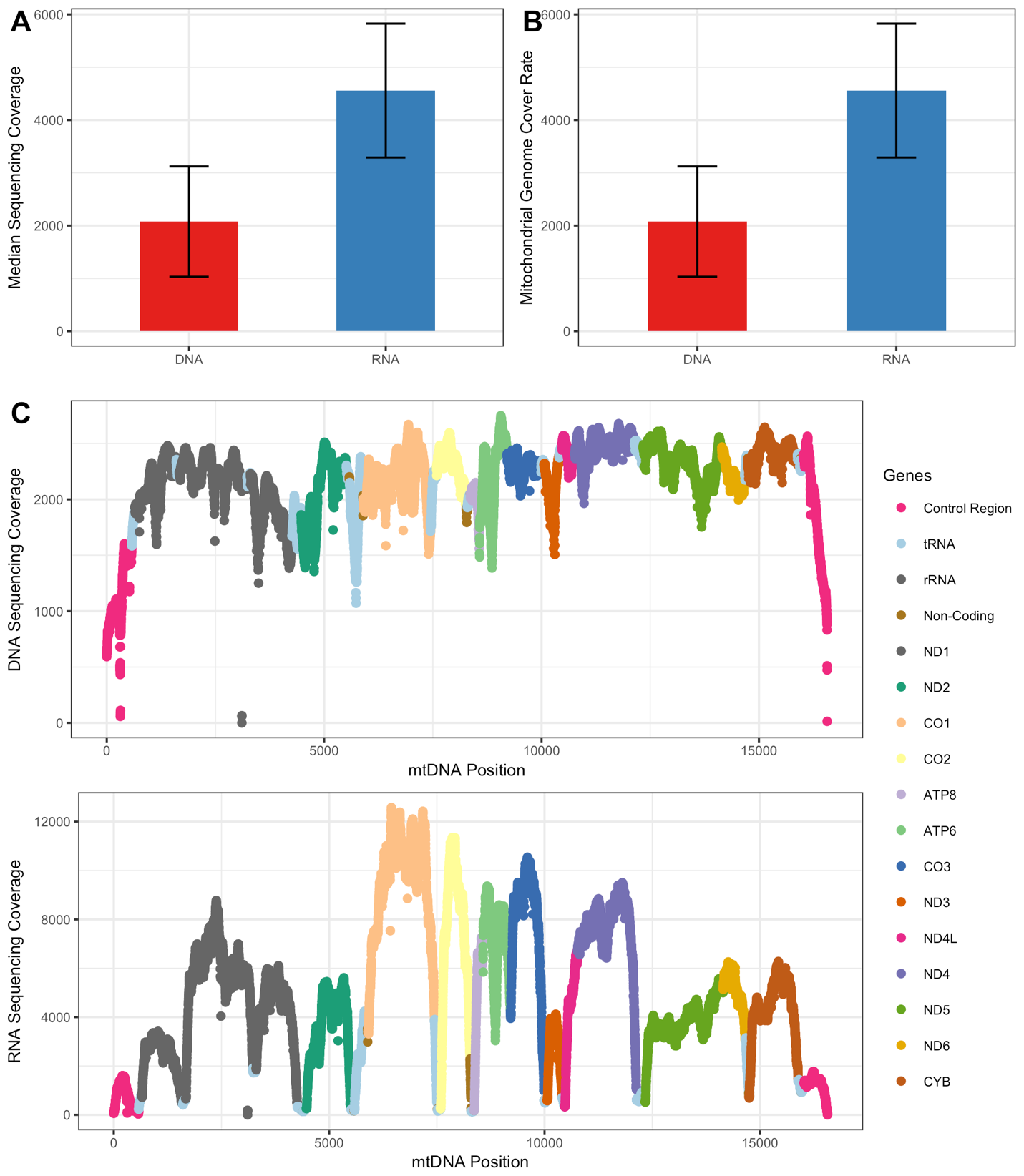


**Figure S2. CADD pathogenic scores of HDLR and non-HDLR heteroplasmies.**

**
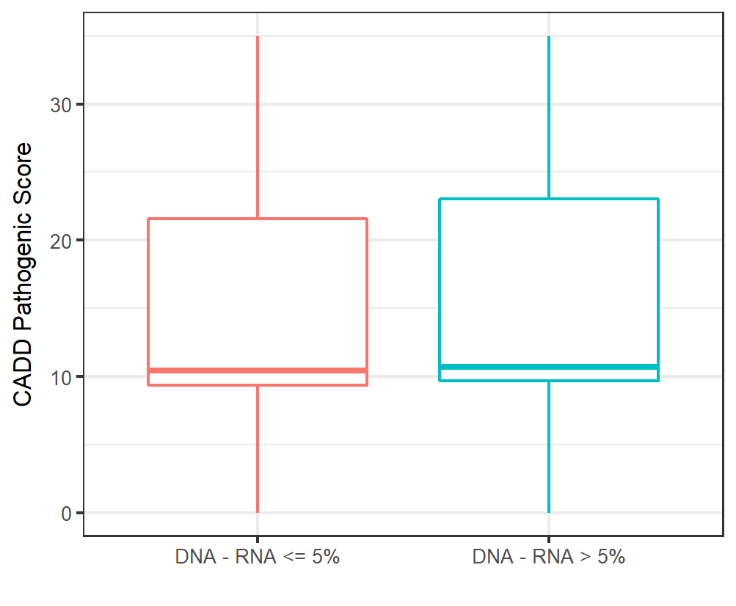
**

**Figure S3. Sanger sequencing trace of heteroplasmy 5794 T>C and 15153 G>A.**

**Table S1. The three previous reported mtRNA editing sites in this dataset. To avoid noise, for each site, we only included the individuals with sequencing coverage > 400 in both DNA and RNA data, and major allele is same as reference allele.**

|  | # of individuals passed filter | # of individuals has heteroplasmy at the given site | # of individuals has edited allele frequency > 1% | Reference allele | Editing allele |
| --- | --- | --- | --- | --- | --- |
| 295 | 319 | 2 | 306 | C | T |
| 2617 | 445 | 0 | 445 | A | T, G, C |
| 13710 | 445 | 0 | 326 | A | T, G |
